# Supplementary material for: Prediction and functional interpretation of inter-chromosomal genome architecture from DNA sequence with TwinC
Source: Nat Commun. 2026 Apr 20;17:5427. doi: 10.1038/s41467-026-72031-5 (PMC13280383; doi:10.1038/s41467-026-72031-5)
Supplement: Supplementary file 2 — Description of Additional Supplementary Files [file 41467_2026_72031_MOESM2_ESM.pdf]

Description of Additional Supplementary Files

Supplementary Data 1. ENCODE accession IDs of heart left and right ventricle Hi-C samples.

Supplementary Data 2. 4DN accession IDs of heart left ventricle Hi-C samples.

Supplementary Data 3. Accession IDs of GM12878 Hi-C and DNA SPRITE Samples.

Supplementary Data 4. List of enriched versus other transcription factors based on the integrated gradients analysis of TwinC with corresponding TF RNA expression in heart left ventricle.

Supplementary Data 5. Cross-validation fold sizes for heart left ventricle.

Supplementary Data 6. Cross-validation fold sizes for heart right ventricle.

Supplementary Data 7. ATAC-seq samples in heart left and right ventricles from the ENCODE portal.

Supplementary Data 8. List of JASPAR transcription factors motifs we used in our analysis.

Supplementary Data 9. List of ChromBPNet transcription factors motifs from cardiomyocytes.

Supplementary Data 10. List of ChromBPNet transcription factors motifs from smooth muscle cells.

Supplementary Data 11. List of ChromBPNet transcription factors motifs from fibroblasts.

Supplementary Data 12. List of ChromBPNet transcription factors motifs from endothelial cells.
