# Supplementary material for: Prediction and functional interpretation of inter-chromosomal genome architecture from DNA sequence with TwinC
Source: Nat Commun. 2026 Apr 20;17:5427. doi: 10.1038/s41467-026-72031-5 (PMC13280383; doi:10.1038/s41467-026-72031-5)
Supplement: Supplementary file 5 — Transparent Peer Review file [file 41467_2026_72031_MOESM5_ESM.pdf]

nature portfolio

Peer Review File

Prediction and functional interpretation of inter-chromosomal genome architecture from DNA sequence with TwinC

Corresponding Author: Professor William Noble

This file contains all reviewer reports in order by version, followed by all author rebuttals in order by version.

Version 0:

Reviewer comments:

Reviewer #1

(Remarks to the Author)
The paper by Jha et al presents an approach for predicting inter-chromosomal DNA contacts using a convolutional neural network, TwinC. While impactful methods to address unknown aspects of inter-chromosomal contacts are useful, TwinC re-introduces the Orca methodology (Zhou, Nat Genetics 2022) and performs faster which is the sole advantage. Here, the authors propose a method addressing some limitations of existing models, particularly in handling trans contact Hi-C data, however, their approach remains far behind other existing technologies to analyze Hi-C datasets. Infeasible approaches, such as the prediction and enrichment of transcription factor binding sites in 100 kb fragments, as well other significant methodological concerns undermine the reliability of the presented results. The paper neither addresses unknown biology and mechanisms of inter-chromosomal contacts, nor does it meet the standard of innovation and accessibility for a broad readership expected for publication in Nature Communications. Below is a detailed assessment:

Major Comments:
1. Test/Train Contamination in Cross-Validation:
The authors employ five-fold cross-validation, dividing the genome into training, validation, and test sets by chromosomes (19/2/2). However, this approach introduces a critical flaw, confounding the entire training process: the test set is indirectly exposed during the training process, as test chromosomes are included in the cross-validation. To ensure a fair evaluation, the test set should be held out entirely from the beginning, leaving the remaining data for cross-validation, which is considered a standard in the field. The model's performance should then be assessed exclusively on this untouched test set. The current design inflates performance metrics by inadvertently training on the test set, leading to unreliable results.

2. Chromosome-Level Splitting Issues:
In each fold, the authors use entire chromosomes for validation and testing. However, inter-chromosomal interactions depend on chromosomal structures, and chromosomes exhibit unique characteristics (e.g., radial arrangements, gene density and transcription differences). This splitting strategy does not generalize well across chromosomes, as patterns from some chromosomes may not apply to others. A more robust evaluation would involve training on subsets of all chromosomes and testing on different datasets, ensuring that the model learns generalizable patterns rather than chromosome-specific artifacts.

3. Hi-C Labeling Methodology:
The authors label trans contacts using a heuristic approach based on replicate consistency. This introduces two issues:
• Potential biases in label generation could propagate into model training.
• A classification approach was chosen over regression, but this decision is insufficiently justified. The authors should compare the proposed method with regression or unsupervised learning approaches for a fairer evaluation.
4. Biological Interpretation:
While the authors claim an AUROC close to 0.80, it is unclear how this translates into meaningful biological insights which are completely missing. Specifically, the results indicate poor true positive rates (e.g., 0.4925 and 0.4430), which are below the expected threshold for binary classification (random baseline: 0.5). This undermines confidence in the practical utility of the model. Even if the predictions are true, despite the methodological inaccuracy, the authors did not validate findings by the means of other technologies.

5. Donor Splitting:

Separating donors into training and testing groups creates another potential bias. Variability between donors (e.g., batch effects) might artificially inflate performance, as the model indirectly learns donor-specific features rather than generalizable biological ones. The authors should repeat the splitting multiple times with random donor assignments to ensure robustness. Moreover, the replicability of trans contacts across individuals remains unexplored.

6. The main text description does not allow to infer how TwinC works. Completely unclear to me is the following: The locus pairs consistently observed to be in contact across multiple donors are assigned a positive class label, whereas those consistently not in contact are assigned a negative class label. TwinC's architecture comprises a 1D convolutional encoder and a 2D convolutional decoder module. Both input sequences pass through the same encoder module to generate latent embeddings that are input to the decoder module. The decoder module then produces the contact score as output. This paired-sequence design allows TwinC to employ shorter input DNA sequences and fewer model parameters.

7. Why did the authors select the Hi-C datasets of the ventricles to train the model. More recent Hi-C datasets with high sequencing depth have been published and are more appropriate for establishing a 'novel' algorithm. Reference is missing to describe the source.

8. The authors cannot infer from the trained model that it correctly predicts any trans contact without applying orthogonal validation strategies (i.e., 4C, imaging, GAM, SPRITE, HiPoreC, etc.).

9. The authors overinterpret their findings by concluding that AA contacts are mediated by sequence-specific elements whilst the other contacts rely on other layers of regulation. Where is the evidence for such a statement when no gene-regulatory elements were analyzed?

10. The attempt to analyze TFBS in 100 kb bins with Jaspar is infeasible because most TFBS would be represented and enrichment analysis is not possible.

Minor Comments:
- Figure 1A Misinterpretation: The claim that trans contacts contribute 20-30% of all DNA interactions is neither clearly supported by Figure 1A, nor by scientific evidence in listing citations. This percentage is highly variable across datasets (up to 50 % in other studies).
- "This restriction is reasonable for cis interactions because chromatin's polymer properties result in most observed cis contacts occurring within a 1 mbp window." The authors do not present evidence for this statement.
- What do sentences like this mean: "Furthermore, generalizing this single-sequence setup to predict trans interactions is nontrivial."
- The following is incorrect since several paper using Hi-C and imaging methodology showed that trans contacts exist and not necessarily rare: "Furthermore, motivated by the extreme sparsity of typical trans contact maps,"
- The following is imprecise and contradictory to the beginning of the introduction where the authors say that current models focus on cis contacts: "Accordingly, TwinC frames trans contact prediction as a classification task with "contact" versus "no contact" classes, in contrast to the regression setup of existing models."
- Model Comparison with Orca:
While the authors briefly compare TwinC to Orca, the evaluation lacks depth. TwinC's supposed advantages (e.g., faster training) are highlighted, but the biological relevance comparisons remain unclear. Comprehensive benchmarking against Orca is essential to validate claims of superiority.
- ATAC-Seq Substitution Experiments:
The logic behind selecting the "top 10%" and "next 30%" loci for accessibility substitution experiments is poorly explained. What biological insights or justifications support these thresholds? Clarifying this rationale would strengthen the relevance of the experiments.
- Activation Function Choice:
The authors employ ReLU, but its limitations (e.g., dead neurons) are evident in this context, particularly given the imbalanced positive/negative label distribution (Negative labels in test sets are substantially higher than positive labels in all experiments). A sigmoid activation function might yield more balanced predictions.
- Parameter Tuning Transparency:
Hyperparameters such as those for Hi-C label thresholds (e.g., i, m, j, n) are described but lack justification or tuning details. The authors should specify how these were optimized and whether sensitivity analyses were performed.

I co-reviewed this manuscript together with another reviewer to provide the listed points. This is part of the Nature Communications initiative to facilitate training in peer review and to provide appropriate recognition for Early Career Researchers.

(Remarks on code availability)
The code and the methodological steps to establish TwinC do not meet established standards. See details in the detailed points raised by the reviewers.

Reviewer #2

(Remarks to the Author)

I co-reviewed this manuscript with one of the reviewers who provided the listed reports. This is part of the Nature Communications initiative to facilitate training in peer review and to provide appropriate recognition for Early Career Researchers who co-review manuscripts.

(Remarks on code availability)
The structure of the code appears well-organized, and all required information, including a README file with instructions for installation and usage, is provided. While I have not run the code myself, it seems to be a usable resource for the community based on the provided documentation.

Reviewer #3

(Remarks to the Author)
The manuscript presents TwinC, a convolutional neural network (CNN) designed to predict trans-chromosomal (trans) DNA interactions using Hi-C datasets. While intra-chromosomal (cis) interactions have been extensively modeled, trans contacts remain underexplored. The authors demonstrate that TwinC effectively predicts trans contacts with high accuracy (AUROC=0.80) using data from heart tissue. Importantly, the model identifies key genomic features such as compartments, chromatin accessibility, transcription factor binding, and G-quadruplexes that contribute to trans contacts. The study addresses a significant gap in our understanding of trans-genome architecture and offers an interpretable neural network approach. The manuscript presents a valuable contribution to understanding trans-genome organization. Addressing the above concerns, particularly the discussion on hyper-parameter validation and latent space optimization, will strengthen the study further.

* The manuscript lacks sufficient discussion on the choice and validation of hyper-parameters used in training the neural network.
* The choice of latent space dimensionality and its effect on model performance is not explored in detail.

The authors should address the lack of hyper-parameter validation and provide a rationale for the chosen architecture, particularly the selection of the latent space. An approach to quantify model performance could involve evaluating the fraction of variation explained by different latent space dimensions. This analysis would provide clarity on whether the current neural network architecture is optimal and whether the latent space effectively captures trans contact features.
Minor Comments:
* Clarify if model performance varies across different tissue types.
* Provide details on the training-validation split and whether cross-validation was performed.

(Remarks on code availability)

Version 1:

Reviewer comments:

Reviewer #3

(Remarks to the Author)
The reviewer finds that the manuscript has improved significantly after revision and hence recommends its publication

(Remarks on code availability)
I didn't check the code

Reviewer #4

(Remarks to the Author)
TwinC is a parameter-efficient binary classifier for trans chromatin interactions using the underlying DNA sequences as the sole inputs.
The model performs similarly to Orca using fewer parameters, out-performs a GC-content baseline, and is a bit worse than the maximum performance estimated based on replicate concordance. Performance is mostly driven by contacts between regions that are both in the A compartment, with BB and A/B pairs having much worse performance. While it is statistically satisfying that TwinC is trained using biological replicates, it is worth pointing out that there are currently relatively few contexts where ~10 replicates are available.
The main biological findings are based on importance scores, which highlight several tissue-specific transcription factors plus G-quadruplexes as sequence features leveraged by the model to predict trans interactions, with the latter being an interesting discovery that might have been impossible or difficult to make without TwinC. While there is some discussion of how the results may relate to nuclear landmarks (e.g., nucleolus) and hubs (e.g., transcriptional factories), there is no analysis of radial positioning or integration with complementary data that would inform on nuclear localization.
I was not an original reviewer of the manuscript. I am focusing my comments regarding the response to reviewers on whether the authors addressed the other reviewers' concerns and recommendations in this revision. The addition of SPRITE and in situ Hi-C cross-technology validations, plus using multiple donor splits in the intact Hi-C analyses, are sufficient to address the comments about inadequate validation. Additions made to the text regarding the methodology and null expectations are clarifying. This is the thorough revision.

It is my policy to sign reviews: Katie Pollard

(Remarks on code availability)

Open Access This Peer Review File is licensed under a Creative Commons Attribution 4.0 International License, which permits use, sharing, adaptation, distribution and reproduction in any medium or format, as long as you give appropriate credit to the original author(s) and the source, provide a link to the Creative Commons license, and indicate if changes were made.
In cases where reviewers are anonymous, credit should be given to 'Anonymous Referee' and the source.
The images or other third party material in this Peer Review File are included in the article's Creative Commons license, unless indicated otherwise in a credit line to the material. If material is not included in the article's Creative Commons license and your intended use is not permitted by statutory regulation or exceeds the permitted use, you will need to obtain permission directly from the copyright holder.
To view a copy of this license, visit https://creativecommons.org/licenses/by/4.0/

Reviewer 1

The paper by Jha et al presents an approach for predicting inter-chromosomal DNA contacts using a convolutional neural network, TwinC. While impactful methods to address unknown aspects of inter-chromosomal contacts are useful, TwinC re-introduces the Orca methodology (Zhou, Nat Genetics 2022) and performs faster, which is the sole advantage. Here, the authors propose a method addressing some limitations of existing models, particularly in handling trans contact Hi-C data; however, their approach remains far behind other existing technologies to analyze Hi-C datasets. Infeasible approaches, such as the prediction and enrichment of transcription factor binding sites in 100 kb fragments, as well other significant methodological concerns undermine the reliability of the presented results. The paper neither addresses unknown biology and mechanisms of inter-chromosomal contacts, nor does it meet the standard of innovation and accessibility for a broad readership expected for publication in Nature Communications. Below is a detailed assessment:

While we appreciate these candid remarks, we respectfully disagree with the characterization of our study. We would like to clarify that the TwinC model is not simply much faster than Orca but is the only practical way to systematically predict trans contacts for a large mammalian genome. We have also developed methods for interpreting our model's behavior, linking its predictions to specific types of sequence elements, and we have demonstrated that the TwinC model generates hypotheses for new biological discovery (e.g., related to the role of G-quadruplexes) in the poorly studied field of trans interactions. Our revised manuscript strengthens and expands these points, showcasing the potential of TwinC to address, for the first time, the fundamental question about trans genome architecture structure/function. We believe this will be of broad interest to the readership of this journal.

1. Test/Train Contamination in Cross-Validation:
The authors employ five-fold cross-validation, dividing the genome into training, validation, and test sets by chromosomes (19/2/2). However, this approach introduces a critical flaw, confounding the entire training process: the test set is indirectly exposed during the training process, as test chromosomes are included in the cross-validation. To ensure a fair evaluation, the test set should be held out entirely from the beginning, leaving the remaining data for cross-validation, which is considered a standard in the field. The model's performance should then be assessed exclusively on this untouched test set. The current design inflates performance metrics by inadvertently training on the test set, leading to unreliable results.

We believe that our current approach is valid. You would be correct if we had run our cross-validation procedure multiple times and selected hyperparameters based on the average performance across the folds. We did not do this, as each fold was validated independently, and we averaged only the resulting performance measures. You are certainly correct that a single train/validation/test split is commonly used these days. However, this is primarily because training many models on massive datasets can be very expensive. Cross-validation is a well-established procedure in statistics and machine learning, dating back many decades (e.g., Picard, Richard; Cook, Dennis (1984). "Cross-Validation of Regression Models." *Journal of the American Statistical Association*. 79 (387): 575–583.) We have clarified this important methodological detail in the revised manuscript to avoid future misunderstandings:

We perform five-fold cross-validation [1] and, in each fold, divide the 23 human chromosomes into training, validation, and test sets of 19, 2, and 2 chromosomes, respectively. We exclude the Y chromosome and mitochondrial DNA from our analysis. The validation set is used for hyperparameter selection for its corresponding fold, and each model is evaluated on its corresponding test set, averaging test set performance across folds.

2. Chromosome-Level Splitting Issues:
In each fold, the authors use entire chromosomes for validation and testing. However, inter-chromosomal interactions depend on chromosomal structures, and chromosomes exhibit unique characteristics (e.g., radial arrangements, gene density and transcription differences). This splitting strategy does not generalize well across chromosomes, as patterns from some chromosomes may not apply to others. A more robust evaluation would involve training on subsets of all chromosomes and testing on different datasets, ensuring that the model learns generalizable patterns rather than chromosome-specific artifacts.

We disagree with this critique. First, it's important to recognize that, because our model takes only sequence

as input, it is not possible to "train on subsets of chromosomes and test on different datasets." The reason is that the model has no way to generalize across biosamples, since the DNA sequence remains fixed. This is why all existing sequence-only models (e.g., Akita and Orca for Hi-C, but also models such as Enformer or Borzoi for RNA-seq prediction) can only be tested on their ability to generalize to new genomic regions within a given biosample. Regarding the first part of the comment above, we do not understand the critique. We agree that "inter-chromosomal interactions depend on chromosomal structures, and chromosomes exhibit unique characteristics"; however, we believe that our results explicitly show that our model does successfully generalize to never-before-seen chromosomes. Thus, the reviewer's claim that "This splitting strategy does not generalize well across chromosomes, as patterns from some chromosomes may not apply to others" appears to be demonstrably incorrect.

3. Hi-C Labeling Methodology:

The authors label trans contacts using a heuristic approach based on replicate consistency. This introduces two issues:

- Potential biases in label generation could propagate into model training.

  We avoided preprocessing steps like the adaptive coarse-graining and smoothing employed by previous sequence-to-Hi-C models, Akita and Orca, because these are likely to introduce biases in the Hi-C data. Instead, we opted to work with standard Knight-Ruiz normalized Hi-C data to minimize bias. It is possible that the reviewer is referring to potential biases due to the randomness in the assignment of donors into training and test sets. As described in more detail below, in our revised manuscript, we have explicitly quantified the effect of this randomness on our results.

  To quantify potential biases in label generation resulting from donor selection, we randomly assigned five donors from the heart left ventricle Hi-C experiments to the label group and the remaining five to the prediction group, repeating this process four times. Subsequently, we trained four TwinC models, one for each donor group used for label assignment. Supplementary Figure 4 shows that the AUROC on the test set is consistent across four different label groups. This result demonstrates that our model's performance is consistent regardless of which five donors were used in the label group.

- A classification approach was chosen over regression, but this decision is insufficiently justified. The authors should compare the proposed method with regression or unsupervised learning approaches for a fairer evaluation.

  As a matter of fact, in Figure 2 of our submitted manuscript, we already compare TwinC with Orca, the previous state-of-the-art method, in a regression setting. Our results show that TwinC, despite being much smaller and faster, is sometimes better than and at least comparable to the Orca model in the regression setting. We have added additional text to the manuscript discussing our motivation for switching to the classification setting.

  The high sparsity of *trans* contacts motivates the switch from the standard, regression-like framework to a classification setting. In the classification setting, we leverage multiple biological replicates to find reproducible contacts shared across replicates and use them as robust positive labels. Similarly, we leverage agreement between biological replicates regarding the absence of contacts to obtain reliable negative labels.

4. Biological Interpretation:

While the authors claim an AUROC close to 0.80, it is unclear how this translates into meaningful biological insights which are completely missing. Specifically, the results indicate poor true positive rates (e.g., 0.4925 and 0.4430), which are below the expected threshold for binary classification (random baseline: 0.5). This undermines confidence in the practical utility of the model.

Here is the exact text from our manuscript: "Similarly, in the heart right ventricle, at the FPR of 0.0482, TwinC's TPR of 0.4430 is only 0.0691 below the upper bound TPR of 0.5121." For a random classifier, the ROC curve lies on the line $y = x$. Hence, at a false positive rate of 0.0482, a random classifier is expected to

achieve a true positive rate of 0.0482. Our model's performance of 0.4430 is thus substantially better than chance. We have added a clarifying sentence to avoid similar misunderstandings:

For a random classifier, the ROC curve lies on the line y = x. Hence, at a false positive rate of 0.0482, a random classifier is expected to achieve a true positive rate of 0.0482.

Even if the predictions are true, despite the methodological inaccuracy, the authors did not validate findings by the means of other technologies.

Several previous studies have established that Hi-C as a technology agrees with other assays, e.g., GAM and SPRITE, despite assay-specific biases (Fiorillo *et al. Nat Methods* 2021; Oksuz *et al. Nat Methods* 2021). In this study, we do not claim that our model finds new trans contacts; rather, we claim that our model can accurately predict observed trans contacts solely on the basis of the DNA sequence. Nonetheless, in response to this request, in the revised manuscript, we now investigate the consistency of our predictions (on test chromosomes) for a model trained using intact Hi-C from the heart left ventricle relative to four replicates of *in situ* Hi-C data from the heart left ventricle.

Furthermore, we evaluate the TwinC model using *in situ* Hi-C data in the heart left ventricle (Supplementary Table 2). Supplementary Figure 3 shows that the TwinC model, which is trained on intact Hi-C, achieves an AUROC of 0.8291 when using *in situ* Hi-C as labels compared to an AUROC of 0.7620 achieved by the GC baseline, highlighting the model's ability to generalize across orthogonal chromatin conformation datasets that differ in their preservation of nuclear architecture.

Moreover, to provide an even more challenging test of TwinC's potential, we train TwinC on *in situ* Hi-C in the GM12878 cell line, a widely studied cell line, and validate using orthogonal DNA SPRITE data in GM12878. The results (summarized below) show that TwinC can also predict trans contacts detected through non-proximity ligation-based approaches. This performance demonstrates TwinC's ability to generalize across Hi-C protocols with distinct experimental biases: intact Hi-C preserves higher-order nuclear architecture such as lamina-associated domains and radial chromatin positioning, while in situ Hi-C emphasizes local interaction patterns but may partially disrupt global nuclear constraints. The model's robustness across these orthogonal representations underscores its capacity to capture transferable features of genome organization.

For orthogonal validation of *trans*-contact predictions made by TwinC, we leveraged *in situ* Hi-C and DNA SPRITE experiments in the GM12878 cell line, a widely used cell line with several available 3D structure measurements (Supplementary Table 3) [2, 3]. *In situ* Hi-C uses the relative frequency of DNA-DNA proximity ligation events for genome-wide detection of pairwise interactions in the nucleus [4]. In contrast, DNA SPRITE uses split-pool recognition of interactions by tag extension for genome-wide detection of higher-order interactions within the nucleus without relying on proximity ligation [3]. However, the DNA SPRITE data can be converted from higher-order interaction clusters to a 2D contact map, and we used a previously generated 2D map [3] for our analysis. First, we trained TwinC using *in situ* Hi-C data in GM12878 using five-fold cross-validation with the same train/validation/test splits as the heart ventricles data (Fig.2A). Subsequently, we evaluated the TwinC model's predictions on the test set chromosomes using DNA SPRITE data as labels. Fig.2B shows that at 1 Mb resolution (the resolution used by [3] for comparison between *in situ* Hi-C and DNA SPRITE) using DNA SPRITE data as labels, the agreement between the two experimental technologies is high (AUROC: 0.9611), and TwinC's predictions are close to this upper limit (AUROC: 0.9336). This result shows that TwinC's predictions generalize across experimental techniques for quantifying nuclear architecture based on fundamentally distinct principles.

5. Donor Splitting:

Separating donors into training and testing groups creates another potential bias. Variability between donors (e.g., batch effects) might artificially inflate performance, as the model indirectly learns donor-specific features rather than generalizable biological ones. The authors should repeat the splitting multiple times with random donor assignments to ensure robustness. Moreover, the replicability of trans contacts across individuals remains unexplored.

We have carried out the proposed experiment with four different donor splits and measured the performance of the TwinC model on labels derived from each donor group. As described in the response to the "Hi-C

Labeling Methodology" section, Supplementary Figure 4 shows that TwinC's performance remains consistent regardless of the donor split.

6. The main text description does not allow to infer how TwinC works. Completely unclear to me is the following: The locus pairs consistently observed to be in contact across multiple donors are assigned a positive class label, whereas those consistently not in contact are assigned a negative class label. TwinC's architecture comprises a 1D convolutional encoder and a 2D convolutional decoder module. Both input sequences pass through the same encoder module to generate latent embeddings that are input to the decoder module. The decoder module then produces the contact score as output. This paired-sequence design allows TwinC to employ shorter input DNA sequences and fewer model parameters.

We are sorry that you had trouble understanding our description. We have revised and expanded the description of the labeling scheme as follows:

The high sparsity of *trans* contacts motivates the switch from the standard, regression-like framework to a classification setting. In the classification setting, we leverage multiple biological replicates to find reproducible contacts shared across replicates and use them as robust positive labels. Similarly, we leverage agreement between biological replicates regarding the absence of contacts to obtain reliable negative labels.

We also expanded the description of the model itself:

Given two input sequences, the TwinC model first employs a 1D convolutional encoder to generate one sequence embedding for each given sequence. We chose 128 *times* 5 for the embedding dimension because it provides a slight performance benefit (Supplementary Figure 2). This encoder can capture local sequence motifs and their interactions, which are often relevant for genomic interactions. The two embeddings from the encoder are combined using outer addition. Outer addition between two N-dimensional tensors produces an $(N + 1)$-dimensional tensor where each element is the sum of one element from each input tensor, with broadcasting over all combinations of indices. The resulting tensor is passed to a 2D convolutional decoder, which integrates spatial interaction patterns between the two input embeddings and subsequently produces the contact score. By independently encoding two input sequences, TwinC can model long-range genomic interactions across arbitrary distances while maintaining a compact parameter footprint. For example, with 6.5 M parameters, TwinC is 3.5-fold smaller than the inter-chromosomal part of the Orca model with 22.5 M parameters. Further architectural details of the TwinC model are described in the Methods section.

7. Why did the authors select the Hi-C datasets of the ventricles to train the model. More recent Hi-C datasets with high sequencing depth have been published and are more appropriate for establishing a 'novel' algorithm. Reference is missing to describe the source.

We selected this particular dataset because it has ten replicates from different individuals, allowing us to measure both technical and biological variability (compared to using multiple replicates of the same immortalized cell line). Please note that the data we used is quite recent and has very high coverage. There is no reference for this data because it is only available via the public ENCODE portal; there is no associated publication yet. The reviewer did not give a specific suggestion of alternative datasets they find of potential interest, but we speculate that they may be referring to the large set of lymphoblastoid Hi-C from 10 cell lines and 6 individuals from (Harris *et al. Nat Comm* 2023). In our revised manuscript, we also trained TwinC on this dataset, which totals 42 billion read pairs and 33 billion contacts, the largest we are aware of. The new text describing this experiment is described above in response to point #4.

8. The authors cannot infer from the trained model that it correctly predicts any trans contact without applying orthogonal validation strategies (i.e., 4C, imaging, GAM, SPRITE, HiPoreC, etc.).

Several previous studies have established agreement between chromosome conformation capture techniques; we are claiming that our model can learn to accurately predict observed trans contacts solely on the basis of the DNA sequence. As mentioned above, in the revised manuscript, we have used publicly available *in situ* Hi-C data from heart ventricles to systematically compare that data to our test intact Hi-C data and to the predictions made by our model. Moreover, to provide evidence that the predictions of trans contacts with TwinC can apply also to chromatin conformation capture independent of proximity-ligation,

we have compared the results from this newly trained model to DNA SPRITE data from the GM12878 lymphoblastoid cell line, which is one of the few such datasets currently available for a sample type also characterized by several replicates of Hi-C. The new text describing this experiment is described above in response to point #4.

9. The authors overinterpret their findings by concluding that AA contacts are mediated by sequence-specific elements whilst the other contacts rely on other layers of regulation. Where is the evidence for such a statement when no gene-regulatory elements were analyzed?

The claim referred to by the reviewer was based on the finding that sequence-only information allows TwinC to predict AA trans contacts with much higher accuracy than BB ones. By exclusion, we hypothesize that epigenetic information plays a more important role in BB trans contacts. We have toned down and clarified our conclusion, indicating that further experiments will be needed to test this hypothesis.

This result suggests that TwinC successfully finds sequence-specific elements that mediate AA contacts but not BB contacts. This lack of sequence-specificity in BB contacts can be due to several reasons. For example, B compartments are enriched in repeat elements and mapping short reads from sequencing experiments to repeat regions is computationally infeasible, potentially resulting in our inability to distinguish contact regions enriched in repeat elements from non-contact regions enriched in repeat elements. Additionally, other layers of epigenomic regulation, such as histone modifications, could be involved. However, further experiments are required to test these hypotheses formally.

10. The attempt to analyze TFBS in 100 kb bins with Jaspar is infeasible because most TFBS would be represented and enrichment analysis is not possible.

We have thought carefully about this analysis, and we are confident that our conclusions are valid. In the current setup, we start by generating per-base importance scores for the top 10% sequence pairs from the test set, ranked by the predicted contact score, using integrated gradients. Subsequently, for every TF and its shuffled control, we find all binding sites within each sequence pair using FIMO, and we take the median importance score of each instance. This process generates two distributions of median importance scores, one for the TF instances and one for the shuffled control instances. We then apply the Wilcoxon-ranksum test to assess the relative magnitude of the median TF importance scores compared to the shuffled control. We repeat this process for each TF in the JASPAR database and apply multiple testing correction to generate the final list of transcription factors implicated in trans contacts. One limitation of our TFBS analysis is that it is conservative in calling TFs enriched in trans contacts due to the limited resolution of trans Hi-C contacts, resulting in a non-comprehensive but statistically valid list of TFs implicated in trans-contacts. The reviewer is correct that, in this analysis, most TFs are represented. Indeed, by design every TF receives a median importance score in the first step of our analysis. However, our analysis does permit for statistical testing of these scores by comparing to the distribution produced by the shuffled control TF.

(Remarks on code availability): The code and the methodological steps to establish TwinC do not meet established standards. See details in the detailed points raised by the reviewers.

This comment is confusing because we could not find the “detailed points raised by the reviewers.” Perhaps we did not receive all the comments from Reviewers 1 and 2.

Reviewer 2

(Remarks on code availability): The structure of the code appears well-organized, and all required information, including a README file with instructions for installation and usage, is provided. While I have not run the code myself, it seems to be a usable resource for the community based on the provided documentation.

We thank the reviewer for this positive assessment.

Reviewer 3

The manuscript presents TwinC, a convolutional neural network (CNN) designed to predict trans-chromosomal (trans) DNA interactions using Hi-C datasets. While intra-chromosomal (cis) interactions have been extensively modeled, trans contacts remain underexplored. The authors demonstrate that TwinC effectively predicts trans contacts with high accuracy (AUROC=0.80) using data from heart tissue. Importantly, the model identifies key genomic features such as compartments, chromatin accessibility, transcription factor binding, and G-quadruplexes that contribute to trans contacts. The study addresses a significant gap in our understanding of trans-genome architecture and offers an interpretable neural network approach. The manuscript presents a valuable contribution to understanding trans-genome organization. Addressing the above concerns, particularly the discussion on hyper-parameter validation and latent space optimization, will strengthen the study further.

The manuscript lacks sufficient discussion on the choice and validation of hyper-parameters used in training the neural network.

In response to this request, we added additional text describing our hyperparameter selection procedure.

We estimated the optimal values for three hyperparameters using the performance on the validation set. First, we optimized the learning rate to achieve the best AUROC on the validation set and fastest convergence of training to be 1×10^{-2} from the set of (1×10^{-1}, 1×10^{-2}, 1×10^{-3}, 1×10^{-4}). Second, we tested five dimensions for the latent space (32×5, 64×5, 128×5, 256×5, 512×5) and observed that all five latent space dimensions resulted in similar predictive performance on the validation set (AUROC: 0.83-0.84, Supplementary Figure 2), with 128×5 latent dimensions resulting in a slight performance advantage. Therefore, we selected 128×5 as our latent space dimension. Third, we tested maximum pooling versus average pooling layers in our model and decided to use max pooling layers in our 1D and 2D convolutional modules based on the predictive performance on the validation set.

The choice of latent space dimensionality and its effect on model performance is not explored in detail. The authors should address the lack of hyper-parameter validation and provide a rationale for the chosen architecture, particularly the selection of the latent space. An approach to quantify model performance could involve evaluating the fraction of variation explained by different latent space dimensions. This analysis would provide clarity on whether the current neural network architecture is optimal and whether the latent space effectively captures trans contact features.

We thank the reviewer for this suggestion. As mentioned in the previous response, we tested five latent space dimensions (32×5, 64×5, 128×5, 256×5, 512×5). We observed that all five latent space dimensions resulted in similar predictive performance on the validation set (AUROC: 0.83-0.84, Supplementary Figure 2), with 128×5 latent dimensions resulting in a slight performance advantage. Therefore, we selected 128×5 as our latent space dimension.

References

[1] Richard R Picard and R Dennis Cook. Cross-validation of regression models. *Journal of the American Statistical Association*, 79(387):575–583, 1984.

[2] Hannah L Harris, Huiya Gu, Moshe Olshansky, Ailun Wang, Irene Farabella, Yossi Eliaz, Achyuth Kalluchi, Akshay Krishna, Mozes Jacobs, Gesine Cauer, et al. Chromatin alternates between a and b compartments at kilobase scale for subgenic organization. *Nature Communications*, 14(1):3303, 2023.

[3] S. A. Quinodoz, N. Ollikainen, B. Tabak, A. Palla, J. M. Schmidt, E. Detmar, M. M. Lai, A. A. Shishkin, P. Bhat, Y. Takei, V. Trinh, E. Aznauryan, P. Russell, C. Cheng, M. Jovanovic, A. Chow, L. Cai, P. McDonel, M. Garber, and M. Guttman. Higher-order inter-chromosomal hubs shape 3D genome organization in the nucleus. *Cell*, 174(3):744–757, 2018.

[4] S. S. P. Rao, M. H. Huntley, N. Durand, C. Neva, E. K. Stamenova, I. D. Bochkov, J. T. Robinson, A. L. Sanborn, I. Machol, A. D. Omer, E. S. Lander, and E. L. Aiden. A 3D map of the human genome at kilobase resolution reveals principles of chromatin looping. *Cell*, 59(7):1665–1680, 2014.
